# *Ixodes ricinus*‐Contact Phase Inhibitor Promotes Functional Recovery and Attenuates Neutrophil Associated Neuroinflammation After Experimental Intracerebral Hemorrhage

**DOI:** 10.1002/cns.71227

**Published:** 2026-10-30

**Authors:** Stéphanie Demoulin, Annelise Letourneur, Florent Lebrun, Nicolas Violle, Denis Vivien, Valérie Pireaux

**Affiliations:** ^1^ Bioxodes SA Gosselies Belgium; ^2^ ETAP‐Lab Caen France; ^3^ Department of Clinical Research Caen University Hospital Caen France

**Keywords:** functional recovery, intracerebral hemorrhage, neuroinflammation, neutrophil extracellular traps, neutrophils, secondary brain injury

## Abstract

**Aims:**

Neutrophil‐associated inflammation contributes to secondary brain injury after intracerebral hemorrhage (ICH). We evaluated whether *Ixodes ricinus* ‐Contact Phase Inhibitor (Ir‐CPI), a selective factor XIa/XIIa inhibitor with reported thromboinflammatory modulatory properties, improves outcome after experimental ICH without worsening bleeding.

**Methods:**

In a collagenase‐induced mouse ICH model, Ir‐CPI was administered as an intravenous bolus followed by a 3‐day infusion. Vehicle animals served as controls and enoxaparin as an anticoagulant comparator. Cohorts evaluated hemorrhage and lesion volumes, endothelial activation, neutrophil‐associated inflammation, and longitudinal functional recovery. Ir‐CPI association with human neutrophils was evaluated in vitro.

**Results:**

Ir‐CPI did not increase hemorrhage or lesion volumes versus PBS through Day 14, whereas enoxaparin showed a higher Day 1 hemorrhage volume than Ir‐CPI. Flow cytometry showed Ir‐CPI association with human neutrophils. In the mouse model, Ir‐CPI produced region‐specific reductions in neutrophil infiltration and NET formation and reduced neuronal degeneration at the injection site and caudal level, without affecting VCAM‐1‐associated endothelial activation. Ir‐CPI accelerated functional recovery, improving neurological scores and sensorimotor asymmetry by Day 5, with restoration of baseline corner test performance by Day 14.

**Conclusion:**

Ir‐CPI improves recovery after experimental ICH without exacerbating bleeding and is associated with reduced neutrophil‐associated secondary brain injury. These findings support its translational potential as a therapeutic strategy for ICH.

## Graphical abstract

*Ixodes ricinus* ‐Contact Phase Inhibitor (Ir‐CPI), a selective FXIa/XIIa inhibitor with reported thromboinflammatory modulatory properties, improves recovery after intracerebral hemorrhage (ICH) without increasing bleeding. By targeting thromboinflammation, Ir‐CPI limits neutrophil infiltration and NET formation, reducing secondary neuronal injury. These effects translate into accelerated functional recovery in experimental ICH.

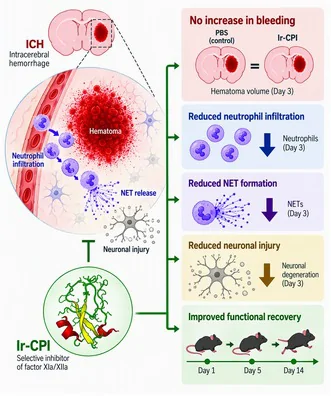

## 1 Introduction

Intracerebral hemorrhage (ICH) is the most severe subtype of stroke, accounting for approximately 10%‐15% of all strokes in high‐income countries yet contributing disproportionately to stroke mortality. Survivors frequently experience persistent neurological deficits and fewer than one in five patients regain functional independence at 6 months [1]. Current acute management focuses on stabilization, blood pressure control, reversal of coagulopathy, prevention of complications, and management of mass effect, but no disease‐modifying pharmacological therapy has consistently improved functional outcome after ICH [2].

Brain injury following ICH reflects both the immediate mechanical consequences of hematoma formation and a secondary injury cascade that evolves over hours to days. Secondary brain injury (SBI) is driven by interconnected processes including neuroinflammation, red blood cell lysis, and thrombin activation, all of which promote blood–brain barrier (BBB) disruption, perihematomal edema (PHE), and neuronal death [3, 4]. PHE development follows a characteristic temporal profile, with rapid expansion during the first 24‐72 h after ICH, making this early period a critical therapeutic window [5].

Neutrophils are among the earliest immune cells recruited after ICH, peaking around Day 3 in experimental models [6, 7]. They contribute to injury through the release of neutrophil extracellular traps (NETs), which amplify inflammation, disrupt BBB integrity and exacerbate neuronal damage [8, 9]. Inhibition of neutrophil recruitment has been shown to reduce brain edema and tissue damage in hemorrhagic stroke models, highlighting neutrophil‐driven inflammation as a promising therapeutic target [10, 11]. NETs were reported to promote neuroinflammation, neuronal damage and brain edema after subarachnoid hemorrhage while pharmacological inhibition of NETs reduced inflammatory damage in mice [12]. Furthermore, a high neutrophil‐to‐lymphocyte ratio was reported to be independently associated with PHE growth and to be a predictor of poor clinical outcomes in ICH patients [13, 14]. In addition, NET components promote coagulation and microvascular dysfunction, linking inflammation and thrombosis [15, 16].

Ir‐CPI ( *Ixodes ricinus* ‐Contact Phase Inhibitor; development code BIOX‐101) is a small protein derived from the saliva of the tick *Ixodes ricinus* that selectively inhibits coagulation factors XIa and XIIa. Ir‐CPI has demonstrated potent antithrombotic efficacy in preclinical models without impairing hemostasis [17, 18]. More recently, Ir‐CPI has been shown to exert anti‐thromboinflammatory effects by reducing neutrophil recruitment in an arteriolar laser‐induced injury mouse model (unpublished data).

Given the significant role of neutrophil‐driven inflammation in SBI, the close link between coagulation and inflammation and the need for therapies that preserve hemorrhagic stability, we tested whether Ir‐CPI could improve recovery after ICH without increasing bleeding. We evaluated Ir‐CPI in a collagenase‐induced mouse model of ICH, a well‐established and reproducible model that induces rapid hematoma formation with peak bleeding within hours of onset [19, 20]. Our findings provide integrated preclinical evidence supporting inhibition of neutrophil infiltration and NET release as a translational strategy to limit secondary brain injury and improve recovery after ICH.

## 2 Methods

### 2.1 Use of Artificial Intelligence

Artificial intelligence (AI) was used solely to assist with language editing and wording refinement during manuscript preparation. No AI tool was used to generate scientific content, analyze data, create figures (except for non‐data illustrations in Figure 1 and the graphical abstract) or draw conclusions. All AI‐assisted text was reviewed and edited by the authors.

**FIGURE 1:**
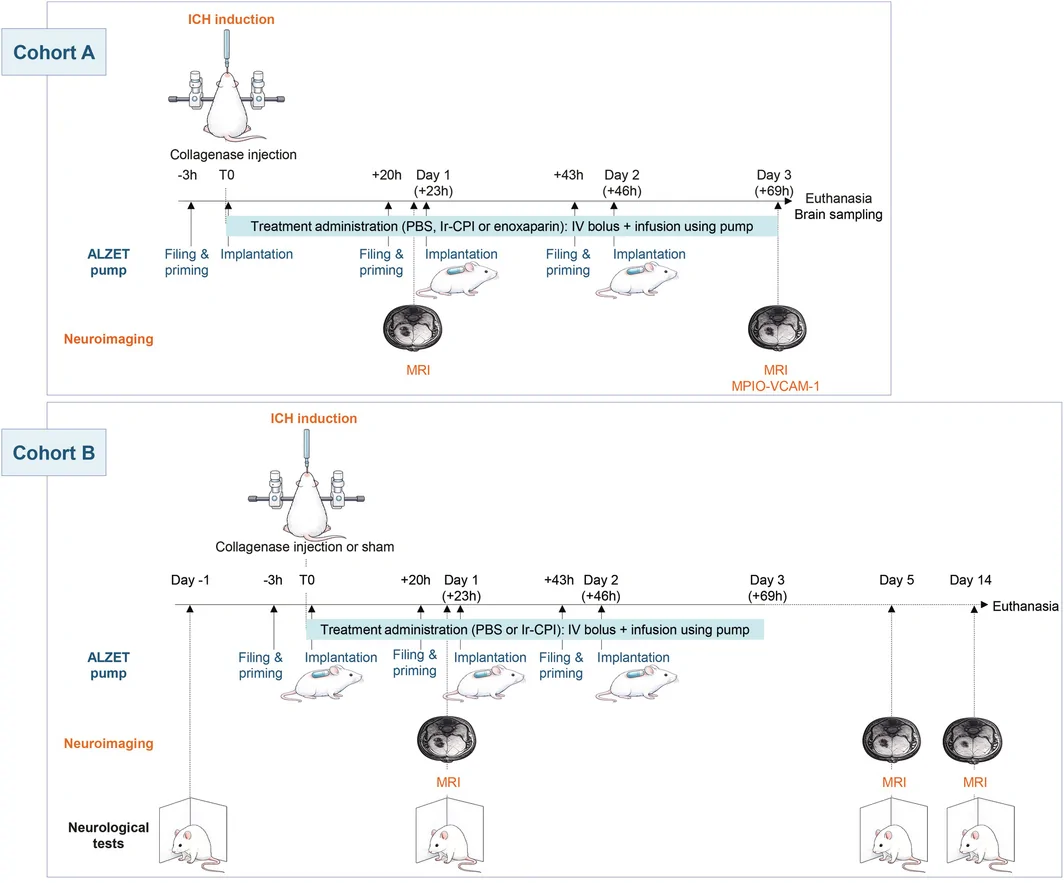
Experimental timelines in the collagenase‐induced ICH model. ICH was induced on Day 0 by stereotaxic injection of bacterial collagenase into the right striatum of adult male OF1 mice. PBS and Ir‐CPI were administered intravenously via a jugular catheter immediately after ICH induction as a bolus followed by continuous infusion for 69 h using implanted osmotic pumps. Enoxaparin was administered as an intravenous bolus followed by continuous saline infusion for 69 h. Pumps were filled and primed 3 h prior to implantation. Sham‐operated mice (Cohort B) underwent identical surgical procedures without collagenase injection and received PBS. Cohort A was designed to assess hemorrhage and lesion evolution and neutrophil‐mediated neuroinflammation during the first 3 days post‐ICH, whereas Cohort B was used to evaluate hemorrhage and lesion evolution as well as functional recovery.

### 2.2 Study Design

Cohort A evaluated hemorrhage and lesion evolution, neuroinflammation, endothelial activation, and neuronal degeneration within the first 3 days after ICH. Cohort B assessed hemorrhage safety, lesion evolution, and functional recovery up to 14 days post‐ICH (Figure 1). Experimental procedures, regimens, randomization, and blinding were consistent across cohorts.

### 2.3 Mice and ICH Induction

Researchers performed all procedures in compliance with the 3Rs and in accordance with European Directive 2010/63/EU. Animal experiments were previously approved (APAFIS#32543 and APAFIS#45240). Adult male OF1 mice (20–25 g) were anesthetized with isoflurane (5.0% for induction and 2.0% for maintenance) in a 70% nitrous oxide/30% oxygen mixture. Body temperature was maintained at 37.5°C using a homeothermic pad connected to a rectal probe. ICH was induced by stereotaxic injection of bacterial collagenase (0.04 IU in 0.5 μL sterile NaCl 0.9%; C2399; Sigma‐Aldrich) into the right striatum using a glass needle tip controlled by a micromanipulator. Injection coordinates were defined relative to Bregma based on a mouse brain atlas [21]. After surgery, mice recovered in their housing cage under surveillance. For peri‐operative analgesia, mice received buprenorphine (Buprecare, Axience, Pantin, France; 0.05 mg/kg, s.c.) on the day of ICH and again on Days 1 and 2.

### 2.4 Treatment Administration

Continuous infusion was achieved using ALZET osmotic pumps connected to a jugular catheter (Figure 1).

Ir‐CPI was administered intravenously, immediately after ICH induction, via a jugular catheter as a bolus of 30.7 mg/kg followed by continuous infusion at 8.3 mg/kg/h for 69 h (see Data S1). Vehicle‐treated mice received PBS as an intravenous bolus, followed by a 69‐h continuous PBS infusion (approximately 8 μL/h). Enoxaparin (Lovenox, Sanofi Aventis), used as an anticoagulant comparator, was administered intravenously as a 20 mg/kg bolus, followed by a continuous saline infusion (NaCl 0.9%) for 69 h. The enoxaparin dose was selected as a pharmacologically active comparator based on prior murine studies demonstrating in vivo antithrombotic efficacy at this dose [22]. Sham‐operated mice in Cohort B underwent identical surgical procedures (including catheterization and pump implantation), but without collagenase injection, and received a PBS bolus followed by continuous PBS infusion for 69 h at approximately 8 μL/h.

### 2.5 MRI Assessment of Hemorrhage and Lesion Volumes

Mice were anesthetized with isoflurane (5.0% for induction; 2.0% for maintenance) in 70% nitrous oxide/30% oxygen. Body temperature was maintained at 37.5°C ± 0.5°C and respiratory rate was monitored throughout imaging to assess anesthetic depth. Hemorrhage and lesion volumes were assessed at 24 h (Day 1) and 69 h (Day 3) post‐ICH for Cohort A and on Days 1, 5 and 14 post‐ICH for Cohort B. MRI was performed using a 7 T Pharmascan system (Bruker BioSpin MRI GmbH) equipped with volume transmit and surface receive coils and operated with Paravision v6 software. Hemorrhage volumes were quantified on T2*‐weighted images as hypointense regions and lesion volumes on T2‐weighted images as hyperintense regions. Volumes were calculated as the sum of areas across slices multiplied by slice thickness. Image analysis was performed blindly using ImageJ v1.53 (NIH) and ITK‐SNAP v3.8.0. Additionally, for Cohort A (Day 3), the rostrocaudal distribution of the hemorrhage was characterized using the 20 consecutive coronal T2*‐weighted MRI slices (0.5‐mm slice thickness). For each animal, rostrocaudal hemorrhage extent was calculated as the number of slices spanning the first to the last slice containing detectable hemorrhagic signal multiplied by the 0.5‐mm slice thickness, that is, (last positive slice – first positive slice + 1) × 0.5 mm. The area‐weighted hemorrhage centroid along the serial MRI stack was calculated as Σ(slice number × hemorrhagic area)/Σ(hemorrhagic area).

### 2.6 Assessment of Endothelial Cell Activation

Researchers assessed endothelial activation in Cohort A on Day 3 by molecular MRI targeting VCAM‐1 using antibody‐conjugated iron particles (see Data S1).

### 2.7 Assessment of Neutrophil Infiltration and Activation

Neutrophil infiltration and NET formation were assessed by immunofluorescence using Ly6G, MPO, and citrullinated histone H3 (H3Cit) co‐staining. One 10‐μm section per animal was analyzed at each of three predefined levels: 600 μm rostral to the injection site, at the injection site, and 600 μm caudal to the injection site. Fluorescence images were acquired using a Leica DM6000 epifluorescence microscope, and hemorrhagic core and perihematomal regions were analyzed using ImageJ v1.53 (NIH) (see Data S1).

### 2.8 Assessment of Neuronal Degeneration

Neuronal degeneration was assessed using Fluoro‐Jade C (FJC) staining (TR‐100‐FJ, Biosensis) according to the manufacturer's instructions. One 10‐μm coronal section per animal was analyzed at each of three predefined stereotaxic levels: 600 μm rostral to the injection site, at the injection site, and 600 μm caudal to the injection site. Images were acquired using a Leica DM6000 epifluorescence microscope and analyzed using ImageJ v1.53 (NIH). FJC‐positive cellular profiles were quantified using a DAPI‐anchored analysis. Detailed quantification procedures are provided in the Data S1.

### 2.9 Neurobehavioral Tests

Functional deficits were assessed in Cohort B at baseline (Day‐1) and on Days 1, 5, and 14 post‐ICH using the corner test and a modified Bederson score [23]. Enoxaparin was not included in Cohort B because of the early hemorrhagic signal observed in Cohort A, which precluded meaningful long‐term behavioral evaluation. Sensorimotor deficits were evaluated using the corner test. Mice were placed between two white Plexiglas walls, 27.5 cm high, arranged at a 30° angle. Each mouse was positioned approximately 20 cm from the corner, facing the apex. Upon entering the corner, the direction of turn was recorded. Ten consecutive trials were performed per session, and the percentage of right turns, ipsilateral to the hemorrhage, was calculated. Global neurological deficits were assessed using a modified Bederson score based on forelimb flexion, resistance to lateral push, and circling behavior. Testing was performed in a three‐walled open‐field apparatus to facilitate animal handling. Scores ranged from 0, indicating no observable deficit, to 3, indicating severe neurological impairment, with higher scores reflecting greater impairment [24]. The scale was extended to scores of 4 for longitudinal spinning and 5 for absence of movement.

### 2.10 Assessment of Ir‐CPI Association With Human Neutrophils

Whole blood was collected from three healthy donors under ethical protocol/amendment IXP‐002 (Belgium; Reg. Nr. B09620097253). Following erythrocyte lysis, leukocytes were washed and incubated with Ir‐CPI (2 μM) for 30 min at 37°C, followed by staining with an AF647‐conjugated anti–Ir‐CPI antibody. Cells were subsequently stained with a panel of lineage markers including CD45, CD3, CD20, CD14, HLA‐DR, CD123, CD56, CD16 and Siglec‐8, together with a viability dye (Table S1). Neutrophils were identified as CD45^+^ viable singlets within the granulocyte FSC/SSC gate, using sequential CD3/CD20, CD14/HLA‐DR and CD123/CD56 gating. Neutrophils were then defined as CD16^+^/Siglec‐8^−^ cells, and Ir‐CPI association was quantified within this gated neutrophil population. Data were acquired using a BD LSR Fortessa X20 flow cytometer and analyzed using FlowJo.

### 2.11 Bias Control, Exclusion Criteria and Statistical Analysis

Animals were allocated to treatment groups using cohort‐specific procedures designed to ensure balanced body weight distribution, and outcome assessments were performed under blinded conditions. Predefined exclusion criteria included technical complications, unsuccessful ICH induction and mortality; in Cohort B, animals with a baseline Bederson score > 0 were excluded from neurological outcome analyses. Final group sizes were: Cohort A—PBS (*n* = 7), Ir‐CPI (*n* = 6), enoxaparin (*n* = 6); Cohort B—sham (*n* = 19), PBS (*n* = 20), Ir‐CPI (*n* = 20). Detailed allocation, blinding, exclusion procedures and sample size justification are provided in the Data S1.

For Cohort A, Day 1 and Day 3 hemorrhage and lesion volumes were analyzed by two‐way repeated‐measures ANOVA with time, treatment and their interaction as fixed effects, with measurements matched within animals, followed by Tukey‐adjusted multiple comparisons. Cohort B longitudinal MRI (PBS vs. Ir‐CPI) and behavioral outcomes (including sham) were analyzed using mixed‐effects models with time, treatment and their interaction as fixed effects, followed by Tukey‐adjusted multiple comparisons. Histological endpoints were analyzed using Kruskal‐Wallis tests followed by Dunn's multiple‐comparisons tests. Day 3 rostrocaudal hemorrhage extent and area‐weighted hemorrhage centroid were compared using exact Kruskal‐Wallis tests. Exploratory associations between lesion volume, inflammatory markers and neuronal degeneration were assessed within treatment groups using two‐tailed Spearman rank correlations. Data are shown as median, interquartile range, range and individual values unless otherwise indicated. Statistical significance was set at *p* < 0.05 (GraphPad Prism v11.1.0).

## 3 Results

### 3.1 Ir‐CPI Preserves Hemorrhagic Stability and Does Not Exacerbate Lesion Evolution After Experimental ICH

Collagenase injection produced a reproducible striatal hemorrhage and an associated lesion. In Cohort A (Figure 2a–c), hemorrhage and lesion volumes showed significant time effects (both *p* < 0.001). Hemorrhage volume also showed a treatment effect (*p* = 0.047), without a time × treatment interaction (*p* = 0.09). On Day 1, hemorrhage volume was higher with enoxaparin than with Ir‐CPI (adjusted *p* = 0.004), whereas Ir‐CPI did not differ from PBS. No between‐group differences were observed on Day 3. Similarly, lesion volume was higher with enoxaparin than with Ir‐CPI on Day 1 (adjusted *p* = 0.030), with no treatment (*p* = 0.09) or time × treatment effect (*p* = 0.26) and no group differences on Day 3.

**FIGURE 2:**
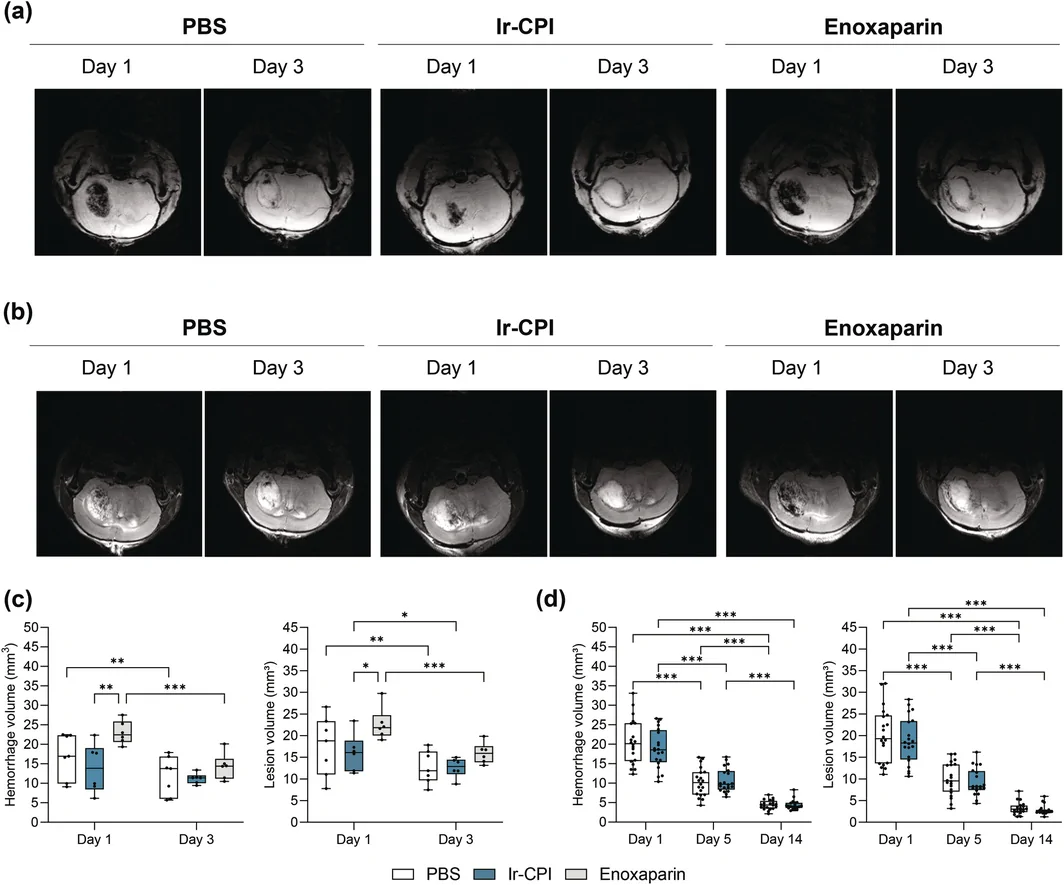
Collagenase‐induced ICH and associated lesion in the right striatum assessed by MRI. Representative hemorrhage (a) and lesion (b) visualization by T2*‐ and T2‐weighted MRI. (c) Hemorrhage volume and total lesion volume on Days 1 and 3 in PBS (*n* = 7), Ir‐CPI (*n* = 6), and enoxaparin (*n* = 6) mice, analyzed by two‐way repeated‐measures ANOVA with measurements matched within animals, followed by Tukey's multiple‐comparisons test. (d) Longitudinal assessment of hemorrhage volume and lesion volume in PBS‐ and Ir‐CPI‐treated animals on Day 1, Day 5 and Day 14 post‐ICH (*n* = 20/group), analyzed using a mixed‐effects model with Tukey's multiple‐comparisons test. Data are presented as box‐and‐whisker plots showing the median (line), interquartile range (box), minimum and maximum values (whiskers) and individual values. *p < 0.05, **p < 0.01, ***p < 0.001.

Day 3 MRI showed no between‐group differences in rostrocaudal hemorrhage extent (median 3.5 mm in all groups; *p* = 0.91) or area‐weighted centroid (*p* = 0.46; Figure S2), supporting anatomical comparability of the predefined histological sampling levels.

In Cohort B (Figure 2d), mixed‐effects analysis showed a significant effect of time on both hemorrhage and lesion volumes (both *p* < 0.001), with no significant treatment effects (*p* = 0.64 and *p* = 0.43, respectively) or time × treatment interactions (*p* = 0.15 and *p* = 0.71, respectively). Tukey‐adjusted within‐group comparisons confirmed progressive decreases in both outcomes over time in PBS‐ and Ir‐CPI‐treated mice (all adjusted *p* < 0.001), whereas no differences were observed between treatment groups on Days 1, 5, or 14 for either hemorrhage or lesion volume (all adjusted *p* ≥ 0.28). Together, these findings indicate that Ir‐CPI preserved hemorrhagic stability and did not exacerbate early or delayed lesion evolution after ICH.

### 3.2 Ir‐CPI Does Not Modulate VCAM‐1‐Associated Endothelial Activation

Relative to historical non‐ICH reference data, ICH was associated with increased VCAM‐1‐associated MPIO signal in both hemispheres. VCAM‐1 labelling volumes were consistently higher in the ipsilateral hemisphere than in the contralateral hemisphere, reflecting a lateralized inflammatory response. No significant differences in VCAM‐1‐associated MPIO signal were observed between groups in either hemisphere or at the whole‐brain level (Figure S1).

### 3.3 Ir‐CPI Shows Strong Association With Neutrophils

Flow cytometry showed strong and consistent Ir‐CPI association with human neutrophils, defined as CD45^+^/CD16^+^/Siglec‐8^−^ cells, with more than 98% Ir‐CPI‐positive cells across donors (Figure 3). In addition, intravital imaging in a murine vein showed spatial association of Ir‐CPI‐associated fluorescence with murine neutrophils (Video S1).

**FIGURE 3:**
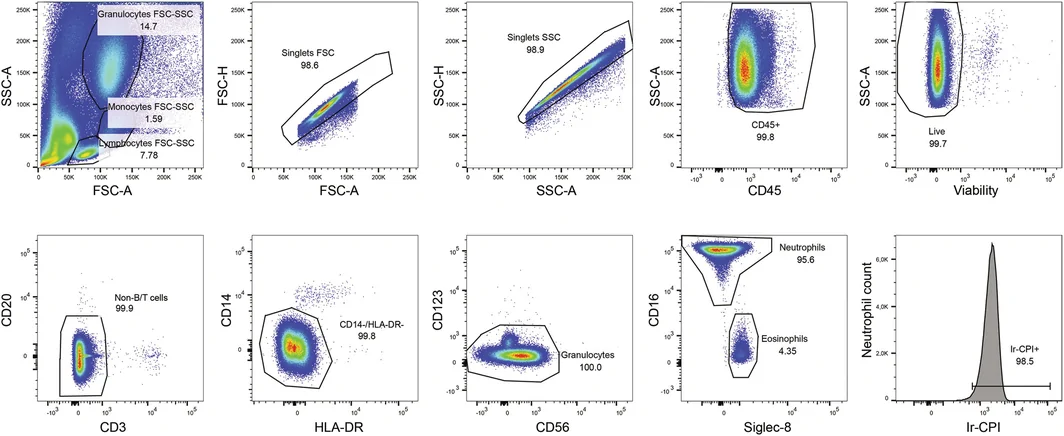
Ir‐CPI shows strong association with neutrophils. Representative flow cytometry gating strategy used to identify human neutrophils and assess Ir‐CPI association with neutrophils. Neutrophils were identified as CD45^+^ viable singlets within the granulocyte FSC/SSC gate, after exclusion of lymphoid and monocytic populations, and were defined as CD16^+^/Siglec‐8^−^ cells. The histogram shows Ir‐CPI association within the gated neutrophil population.

### 3.4 Ir‐CPI Reduces Neutrophil Infiltration and NET Release in the Hemorrhagic Brain

On Day 3 post‐ICH, marked inflammatory infiltration was observed in PBS mice. MPO^+^ cell counts were similar across groups and regions (Figure 4a), consistent with MPO reflecting broader myeloid activity rather than neutrophil‐specific infiltration [25]. In contrast, Ly6G staining revealed pronounced neutrophil accumulation in PBS mice (Figure 4b). Ir‐CPI significantly reduced Ly6G^+^ neutrophils at the rostral level compared with PBS (*p* = 0.01), with a non‐significant reduction at the injection site (*p* = 0.09). When averaged across the three sampled levels, Ly6G^+^ counts were lower in Ir‐CPI‐treated mice than in PBS controls (*p* = 0.08), suggesting an overall attenuation of neutrophil recruitment.

**FIGURE 4:**
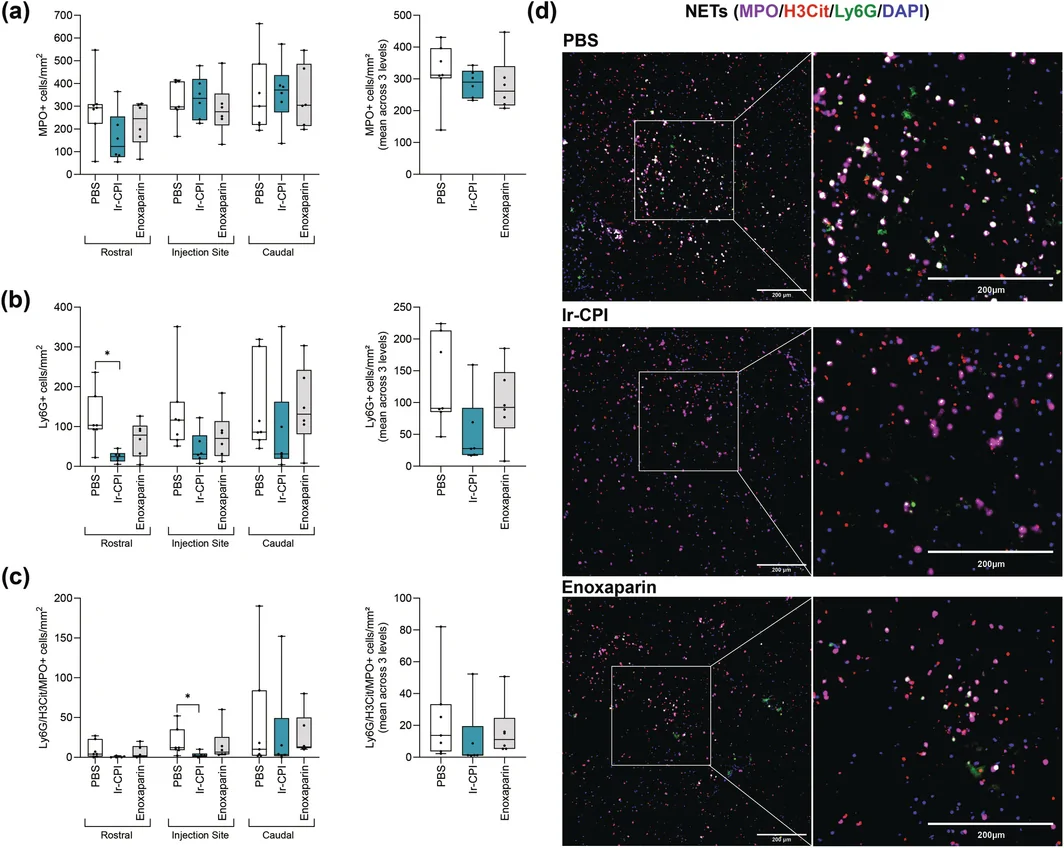
Inflammatory cells, neutrophil infiltration, and NET release after ICH assessed by immunofluorescence. Quantification was performed on Day 3 following collagenase‐induced ICH. (a) MPO^+^ cell density, (b) Ly6G^+^ cell density, and (c) Ly6G^+^/H3Cit^+^/MPO^+^ (NET‐releasing) neutrophil density were quantified at the rostral level (600 μm rostral to the injection site), at the injection site, and at the caudal level (600 μm caudal to the injection site) in the ipsilateral hemisphere. Mean cell densities across the three sampled levels were calculated per mouse. Data are presented as box‐and‐whisker plots showing the median (line), interquartile range (box), minimum and maximum values (whiskers), with individual data points shown. Statistical comparisons were performed using the Kruskal‐Wallis test followed by Dunn's multiple comparisons test (*p < 0.05). Group sizes: PBS (*n* = 7), Ir‐CPI (*n* = 6), enoxaparin (*n* = 6). (d) Representative images demonstrating co‐localization of Ly6G, H3Cit, and MPO in neutrophils within the hemorrhagic area (scale bar: 200 μm).

NET analysis (Ly6G/H3Cit/MPO) showed abundant NET‐releasing neutrophils in PBS mice (Figure 4c,d). Ir‐CPI significantly reduced NET‐releasing neutrophils at the injection site (*p* = 0.03), with a non‐significant reduction at the rostral level (*p* = 0.08). When averaged across the three sampled levels, Ir‐CPI‐treated mice consistently showed lower NET counts than PBS‐treated mice, indicating a global trend toward reduced NET release despite regional variability.

No significant correlation was observed between lesion volume and inflammatory markers in Ir‐CPI mice on Day 3 (Table S2), suggesting that the anti‐inflammatory effects of Ir‐CPI may not be solely attributable to differences in lesion size.

Enoxaparin did not significantly modify neutrophil infiltration or NET release compared with PBS in any region.

Overall, Ir‐CPI attenuates neutrophil‐associated inflammation after ICH, with significant reductions observed at the predefined rostral sampling level for Ly6G^+^ neutrophils and at the injection site for NET‐releasing neutrophils.

### 3.5 Ir‐CPI Attenuates Neuronal Degeneration Following ICH

Neuronal degeneration was assessed on Day 3 using the DAPI‐anchored FJC analysis (Figure 5). The percentage of DAPI‐positive profiles classified as FJC‐positive was significantly lower in Ir‐CPI‐treated mice than in PBS controls at the injection site (Dunn‐adjusted *p* = 0.0430) and at the caudal level (Dunn‐adjusted *p* = 0.0403), with no difference at the rostral level (Figure 5b). At the caudal level, FJC positivity was numerically lower with Ir‐CPI than with enoxaparin, although this comparison did not reach statistical significance (Dunn‐adjusted *p* = 0.072). DAPI‐positive profile density did not differ significantly among treatment groups at any analyzed level (Figure 5c).

**FIGURE 5:**
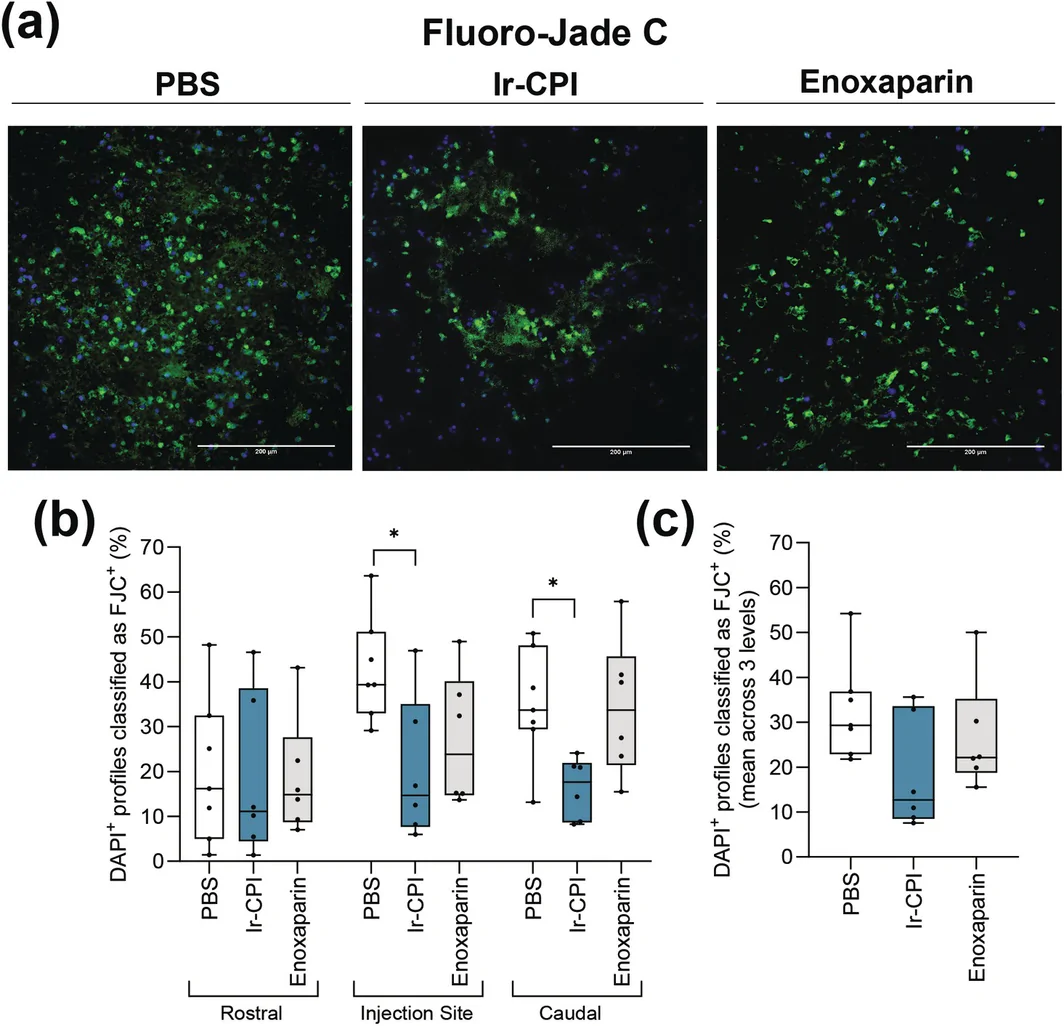
Neuronal degeneration after ICH assessed by Fluoro‐Jade C staining. Neuronal degeneration was assessed on Day 3 using a DAPI‐anchored FJC quantification method. (a) Representative microscopy images (FJC, green; DAPI, blue; scale bar: 200 μm). (b) Percentage of DAPI^+^ profiles classified as FJC^+^ at the rostral level, injection site, and caudal level. (c) Mean percentage of DAPI^+^ profiles classified as FJC^+^ across the three sampled levels for each animal. Statistical comparisons were performed using the Kruskal‐Wallis test followed by Dunn's multiple‐comparisons test where applicable (*p < 0.05). PBS, *n* = 7; Ir‐CPI, *n* = 6; enoxaparin, *n* = 6.

### 3.6 Ir‐CPI Accelerates Functional Recovery After ICH

Functional recovery was evaluated in an independent cohort using the corner test and the Bederson neurological score from baseline (Day‐1) to Day 14 post‐ICH (Figure 6). At baseline, all groups showed comparable neurological status, with Bederson scores of 0 and corner test values close to 50%, confirming equivalent pre‐injury function.

**FIGURE 6:**
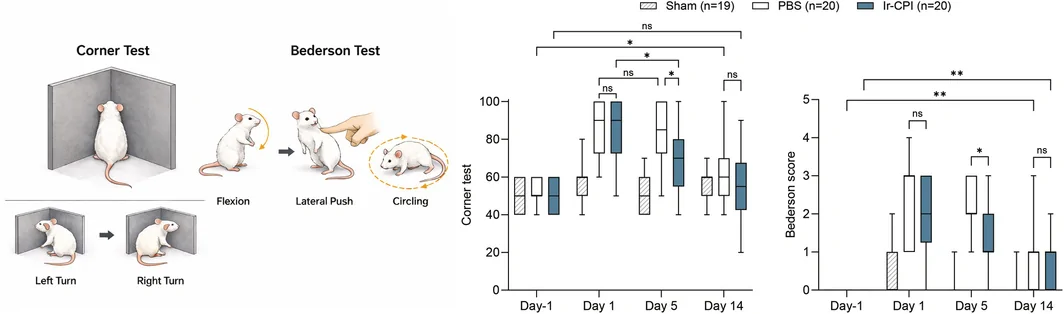
Functional outcome assessment from baseline to Day 14 after ICH induction. Functional outcome was assessed in an independent cohort of mice subjected to collagenase‐induced ICH using the corner test and the Bederson neurological score at baseline (Day‐1) and on Days 1, 5, and 14. Sham‐operated animals underwent identical procedures without collagenase injection. Data are presented as box‐and‐whisker plots showing the median (line), interquartile range (box), and minimum and maximum values (whiskers). Statistical analysis was performed using mixed‐effects modeling followed by Tukey's multiple comparisons test (*p < 0.05, **p < 0.01; ns, not significant). For clarity, statistical comparisons involving the sham group are not displayed in the figure, as the primary objective was to evaluate treatment effects between ICH groups; however, sham animals were included in the statistical model.

ICH induced marked neurological deficits on Day 1. Bederson scores increased significantly in both PBS‐ and Ir‐CPI‐treated mice compared to sham (PBS 2.263 vs. Sham 0.474, *p* < 0.001; Ir‐CPI 2.050 vs. Sham 0.474, *p* < 0.001), with no difference between ICH groups. Similarly, both ICH groups showed pronounced sensorimotor asymmetry in the corner test compared with sham animals (PBS and Ir‐CPI: 86.5% vs. 56.84%; both *p* < 0.001), indicating comparable initial injury severity.

During the subacute phase, sham animals showed stable performance over time and remained significantly less impaired than ICH groups on Day 5 (both *p* ≤ 0.003), indicating persistent hemorrhage‐induced deficits. By contrast, differences between groups emerged on Day 5. By Day 5, PBS animals continued to exhibit severe deficits, whereas Ir‐CPI mice showed significant improvement. Bederson scores were significantly lower in the Ir‐CPI group compared to PBS (1.550 vs. 2.211; mean difference 0.6605, 95% CI 0.02996–1.291; *p* = 0.04). Likewise, corner test performance was significantly improved under Ir‐CPI treatment (69.5% vs. 83.5% in PBS; mean difference 14.0, 95% CI 0.65–27.35; *p* = 0.04).

Importantly, within‐group analyses further highlighted divergent recovery kinetics. Ir‐CPI mice demonstrated significant improvement between Day 1 and Day 5 in the corner test (*p* = 0.01), whereas PBS animals did not. Thus, Ir‐CPI not only reduced deficit severity on Day 5 but also accelerated early recovery. Sham animals showed only minor fluctuations over time and no sustained deficits, consistent with the absence of hemorrhage.

By Day 14, Ir‐CPI‐ and PBS‐treated mice no longer differed significantly in either test. However, recovery trajectories remained distinct: in the corner test, Ir‐CPI‐treated mice returned to baseline performance, whereas PBS‐treated mice remained significantly impaired. Bederson scores indicated mild residual deficits in both ICH groups compared with baseline (both *p* ≤ 0.004).

Overall, Ir‐CPI did not affect initial injury severity but significantly accelerated early neurological recovery and promoted more complete restoration of sensorimotor function after ICH.

## 4 Discussion

This study identifies Ir‐CPI as a hemorrhage‐safe therapeutic candidate that improves functional recovery after experimental ICH and attenuates neutrophil‐associated secondary brain injury.

Preservation of hemorrhagic stability is central to the therapeutic relevance of these findings. Acute ICH remains challenging because anticoagulant strategies may worsen bleeding, as illustrated by a significantly higher Day 1 hemorrhage volume with enoxaparin than with Ir‐CPI in the murine model. Ir‐CPI, which selectively inhibits factors XIa/XIIa, did not increase hemorrhage volume relative to PBS at any assessed time point. This profile is consistent with prior evidence that contact/intrinsic pathway inhibition can reduce thrombosis while preserving hemostasis [17].

Secondary brain injury is now recognized as a major determinant of outcome after ICH and represents a critical therapeutic target beyond the initial hemorrhagic insult. Perihematomal edema expansion, neuroinflammation, and BBB disruption contribute substantially to neurological deterioration after ICH [3, 26]. In this context, the early inflammatory response is increasingly viewed as a modifiable driver of injury rather than a passive consequence of hemorrhage. Neutrophils, which rapidly infiltrate the injured brain and peak around Day 3 post‐ICH, play a key role in this process [6]. In line with clinical observations linking an elevated neutrophil‐to‐lymphocyte ratio with poor outcomes [13, 14], we observed marked neutrophil infiltration and NET release in the hemorrhagic region on Day 3 post‐ICH. Ir‐CPI produced significant region‐specific reductions in neutrophil recruitment and NET release, supporting a role in modulating early neutrophil effector mechanisms that are known to exacerbate SBI.

Flow cytometry demonstrated a strong association of Ir‐CPI with human neutrophils. Although this was not quantitatively assessed in murine neutrophils, intravital imaging of a murine vein showed spatial association of the Ir‐CPI‐associated signal with murine neutrophils (Video S1). Together, these findings support an association of Ir‐CPI with neutrophils, although the molecular partner involved remains unknown and warrants further investigation.

NETs have emerged as key mediators linking inflammation and coagulation in both ischemic and hemorrhagic strokes. Beyond their pro‐inflammatory effects, NET components provide procoagulant surfaces that promote thrombin generation and the formation of dense, lysis‐resistant clots [16, 27]. This thrombo‐inflammatory amplification loop is increasingly recognized as a contributor to microvascular dysfunction and tissue injury. By selectively inhibiting FXIIa and FXIa, Ir‐CPI may disrupt this coagulation‐inflammation interface and limit NET‐driven amplification of damage. This mechanism is distinct from direct inhibition of the kallikrein‐kinin pathway. Ir‐CPI does not inhibit preformed kallikrein or reduce bradykinin generation once the contact system is activated (unpublished data), suggesting that the observed effects may involve upstream FXIIa/FXIa inhibition, potentially limiting downstream thrombo‐inflammatory amplification, together with a direct effect on neutrophils. The relative contribution of these mechanisms remains to be determined.

After ICH, damage‐associated molecular patterns and blood‐derived factors rapidly activate the endothelium, inducing the expression of adhesion molecules such as VCAM‐1, which promote neutrophil adhesion and transendothelial migration [28]. In our model, Ir‐CPI did not modify VCAM‐1‐associated endothelial activation, suggesting that the reduction in neutrophil recruitment is unlikely to be explained solely by changes in VCAM‐1‐associated endothelial activation.

Ir‐CPI may act at both vascular and local sites after ICH. Its association with circulating and adherent neutrophils supports an intravascular interaction, while the vascular and blood–brain barrier disruption induced by collagenase may allow Ir‐CPI to reach the hemorrhagic environment. These mechanisms are therefore not mutually exclusive and may contribute jointly to the reduction in neutrophil recruitment and NET release observed after treatment.

Neuronal death after ICH is driven by a convergence of mechanical injury, excitotoxicity, oxidative stress, and inflammatory signaling [4]. Neutrophil‐derived mediators, including NET‐associated histones, can contribute to secondary neuronal injury. Although our study design does not allow us to establish direct causality, the concordance between reduced neutrophil activation, decreased NET release, and reduced FJC positivity is consistent with attenuated secondary neuronal degeneration following Ir‐CPI treatment. Notably, at the caudal level, neuronal degeneration was numerically lower with Ir‐CPI than with enoxaparin, whereas enoxaparin showed values comparable to PBS. This pattern suggests that the attenuation of neuronal degeneration may not be attributable to anticoagulation alone.

Exploratory correlation analyses showed no significant association between neutrophil infiltration, NET release, or MPO‐positive cell burden and lesion volume in Ir‐CPI‐treated mice (Table S2), suggesting that the anti‐inflammatory effects of Ir‐CPI may not be solely secondary to differences in lesion severity. Likewise, no significant associations were identified between these inflammatory markers and neuronal degeneration within individual treatment groups (Table S3). This suggests that inter‐animal variation in neuronal degeneration was not closely predicted by these inflammatory markers alone; however, given the small group sizes and exploratory nature of the analyses, moderate associations cannot be excluded.

Functional recovery is the most clinically relevant outcome in hemorrhagic stroke, and many experimental interventions that improve histological markers fail to produce behavioral gains. In an independent cohort followed longitudinally, Ir‐CPI significantly improved sensorimotor asymmetry and neurological deficits, with accelerated recovery between Days 1 and 5 and more complete restoration of fine sensorimotor performance (corner test) by Day 14. These findings are consistent with prior reports showing that attenuation of neutrophil recruitment or activation, as well as reduction in the number of neurodegenerative cells, can improve functional outcome after experimental ICH [6, 11, 29].

Intravenous administration of Ir‐CPI is compatible with the general framework of current acute stroke care, in which systemic therapies are routinely initiated soon after diagnosis [30]. This suggests that Ir‐CPI could be integrated into established time‐sensitive protocols, alongside a care bundle, without requiring major structural changes in emergency care organization. In addition, an intravenously delivered agent allows rapid achievement of therapeutic plasma concentrations and flexible titration or discontinuation in response to clinical and radiological monitoring, which is particularly relevant in a setting where hemorrhagic stability must be preserved.

In the present study, Ir‐CPI was administered as a 3‐day infusion covering the early phase of ICH, including hematoma expansion, PHE growth and peak neutrophil infiltration. Despite treatment discontinuation, functional benefits persisted up to Day 14, with accelerated neurological recovery and improved sensorimotor performance. These findings align with previous studies showing that short‐term modulation of early secondary injury cascades can have durable effects on recovery. Although substantial spontaneous recovery occurs within the first 2 weeks in mice [31], the persistence of treatment‐related benefits on Day 14 suggests that Ir‐CPI may favorably modify the early injury milieu and influence longer‐term outcomes.

The collagenase‐induced ICH model offers key advantages, including reproducible hemorrhage, robust SBI and persistent neurological deficits, but it does not fully recapitulate human ICH [20, 32]. Unlike spontaneous ICH, which usually results from focal rupture of diseased small arteries, collagenase causes diffuse vascular disruption at the injection site and may amplify local inflammation and blood–brain barrier injury [32, 33]. This study included only male mice, precluding assessment of sex‐specific responses. Given reported sex differences in experimental and clinical ICH outcomes [34, 35], future studies of Ir‐CPI should incorporate both sexes and models that better reflect human vascular pathology, such as hypertensive or amyloid angiopathy backgrounds.

In addition, histological quantification of inflammatory markers and neuronal degeneration was performed on one 10‐μm section at each of three predefined stereotaxic levels. This standardized regional sampling strategy does not constitute an exhaustive volumetric assessment of the inflammatory response or neuronal degeneration throughout the entire hematoma. Despite these limitations, these data provide strong preclinical support for Ir‐CPI as a potential therapeutic strategy targeting thrombo‐inflammation in ICH. Given the lack of effective disease‐modifying treatments and the central role of neutrophils and NETs in SBI, selective modulation of this axis without increasing bleeding risk represents a promising approach.

## Author Contributions

S.D. critically reviewed the data, performed the statistical analyses, prepared the figures and wrote the original draft of the manuscript. A.L., N.V., and F.L. contributed to the conceptualization of the study and provided scientific supervision. D.V. contributed to study design and provided scientific advice. V.P. supervised the study management, contributed to the conceptualization of the study and critically reviewed the data. All authors reviewed and approved the final manuscript.

## Funding

This work was financially supported by Bioxodes SA. No specific grant was associated with this funding.

## Ethics Statement

All procedures were performed in accordance with the European Directive 2010/63/EU. The French Ministry of Higher Education and Research approved the animal experiments (APAFIS#32543 and APAFIS#45240). Human blood was collected from healthy donors under ethical protocol/amendment IXP‐002 (Belgium; Reg. Nr. B09620097253).

## Conflicts of Interest

S.D. and V.P. are employees of Bioxodes SA. The other authors declare no conflicts of interest.

## Supporting information


**Figure S1:** VCAM‐1‐associated endothelial activation in the brain on Day 3 following collagenase‐induced intracerebral hemorrhage in PBS‐, Ir‐CPI‐ and enoxaparin‐treated mice.
**Figure S2:** Rostrocaudal distribution of the hemorrhage on Day 3 after ICH.

**Data S1:** Supplementary Methods, including osmotic pump preparation and implantation, assessment of endothelial cell activation, neutrophil infiltration and activation, neuronal degeneration, intravital microscopy, randomization, blinding, exclusions and sample size.

**Video S1:** In vivo association of Ir‐CPI with murine neutrophils. Representative intravital microscopy video showing Ir‐CPI‐A488 (green) and Ly6G^+^ neutrophils labeled with anti‐Ly6G‐PE (displayed in blue) within a mouse vein. Ir‐CPI‐associated signal is observed in spatial association with circulating/adherent neutrophils over time. Scale bar: 10 μm.

**Table S1:** Antibodies used in flow cytometry to assess the association of Ir‐CPI with human neutrophils.
**Table S2:** Exploratory correlation analyses between lesion volume and inflammatory markers on Day 3 following collagenase‐induced intracerebral hemorrhage in PBS‐, Ir‐CPI‐, and enoxaparin‐treated mice.
**Table S3:** Exploratory correlations between inflammatory markers and neuronal degeneration on Day 3 following collagenase‐induced intracerebral hemorrhage in PBS‐, Ir‐CPI‐, and enoxaparin‐treated mice.

## Data Availability

The data that support the findings of this study are available on request from the corresponding author.
