# Supplementary material for: Ixodes ricinus‐Contact Phase Inhibitor Promotes Functional Recovery and Attenuates Neutrophil Associated Neuroinflammation After Experimental Intracerebral Hemorrhage
Source: CNS Neurosci Ther. 2026 Oct 30;32(11):e71227. doi: 10.1002/cns.71227 (PMC13722891; doi:10.1002/cns.71227)
Supplement: Supplementary file 1 [file CNS-32-e71227-s002.docx]

**Supplementary Methods**

**Osmotic Pump Preparation and Implantation**

ALZET osmotic pumps (model 2001D) were used for continuous infusion [1]. Pumps were modified with a dedicated kit to replace metallic components and ensure MRI compatibility, filled under sterile conditions, weighed before and after filling and primed for 3 h at 37°C in sterile 0.9% saline prior to implantation. Catheterization, pump implantation and ICH surgery were performed under gaseous anesthesia with isoflurane (5.0% for induction; 2.0% for maintenance) in 70% nitrous oxide/30% oxygen. Body temperature was maintained at 37.5°C using a homeothermic pad connected to a rectal probe. Pumps were implanted subcutaneously in mice bearing a jugular vein catheter. Following ICH induction and intravenous bolus administration, the primed pump was connected to the catheter and implanted in the interscapular region. Pumps were replaced every 23 h under the same anesthesia regimen. Local 4% lidocaine (Instillagel, Farco-Pharma GmbH) was applied to the wound before reopening and after closure. Removed pumps were weighed to confirm treatment delivery. For perioperative analgesia, mice received buprenorphine (Buprecare, Axience, Pantin, France; 0.05 mg/kg, s.c.) on the day of ICH and again on Days 1 and 2.

**Assessment of Endothelial Cell Activation**

Endothelial activation was assessed in mice from Cohort A by molecular MRI targeting vascular cell adhesion molecule-1 (VCAM-1) using anti-VCAM-1-conjugated micro-sized particles of iron oxide (MPIOs) as previously described [2]. MPIOs (Dynabeads MyOne Tosylactivated, diameter 1 µm) were covalently conjugated under sterile conditions with monoclonal rat anti-mouse VCAM-1 antibodies (anti-CD106, clone 429), washed, blocked with 0.5% bovine serum albumin and stored at 4°C until use (≤7 days). Each batch was validated in positive-control mice by intracerebral lipopolysaccharide injection (1 µg; *Escherichia coli* O111:B4; Sigma-Aldrich Merck). On Day 3 post-ICH, mice received an intravenous injection of VCAM-1-conjugated MPIOs. MPIO binding to endothelial VCAM-1 induced focal signal voids on T2*-weighted MRI. Pre- and post-injection T2* sequences were acquired and VCAM-1-positive signal was quantified after whole-brain masking and hemispheric segmentation using a common threshold on normalized images. Results are expressed as VCAM-1-positive volume per hemisphere. Image analysis was performed using ImageJ v1.53 (NIH) and ITK-SNAP v3.8.0. VCAM-1-associated MPIO signal and histological endpoints were analyzed using Kruskal-Wallis tests followed by Dunn’s multiple-comparisons tests. Historical non-ICH data obtained using the same VCAM-1-targeted molecular MRI approach were used as contextual reference values and were not included in the statistical comparisons between treatment groups.

**Assessment of Neutrophil Infiltration and Activation**

For terminal tissue collection on Day 3, mice from Cohort A received buprenorphine (0.05 mg/kg, s.c.) prior to the procedure and were deeply anesthetized with isoflurane (5.0% in 70% nitrous oxide/30% oxygen). Mice were then transcardially perfused with heparinized saline (7 mL/min) followed by 10 mL of 4% formaldehyde at the same flow rate. Brains were collected, frozen and embedded in cryomatrix embedding resin (6769006, Epredia) and stored at -80°C until use. Coronal brain sections (10 µm) were prepared at -20°C, mounted on polylysine‑coated slides and stored at -80°C until immunofluorescence analysis. One 10-µm section at each of three predefined coronal levels per animal was selected for immunostaining, corresponding to three sections per animal in total: the collagenase injection level (+0.5 mm from Bregma), a level 600 µm rostral to the injection site (+1.1 mm from Bregma), and a level 600 µm caudal to the injection site (−0.1 mm from Bregma). These fixed stereotaxic levels were used to provide standardized regional sampling and allow anatomically comparable between-group analyses. Concomitant staining of Ly6G, citrullinated histone H3 (H3-Cit), myeloperoxidase (MPO) and DNA (DAPI) was performed to evaluate inflammatory cells, neutrophil infiltration and activation leading to NET release. Frozen slides were thawed at room temperature (RT) for 30 minutes, rinsed three times in PBS (pH 7.4) and carefully dried. Slices were then incubated overnight at RT in a humidified dark chamber with a mix of primary antibodies diluted in PBS-Triton X-100 0.25%: goat anti-mouse MPO (1:200; AF3667, R&D Systems), rabbit anti-mouse H3-Cit (1:200; ab5103, Abcam) and rat anti-mouse Ly6G (clone 1A8; 1:500; 60031, Stemcell Technologies). The following day, slides were rinsed three times in PBS and incubated for 1h30 at RT with a mix of secondary antibodies diluted in PBS-Triton X-100 0.25%: donkey anti-goat coupled to Alexa Fluor 647, anti-rabbit coupled to Cy3 and anti-rat coupled to Alexa Fluor 488 (all 1:800; Jackson ImmunoResearch). Slides were then rinsed three times in PBS, air-dried and coverslipped with antifade mounting medium containing DAPI (Fluoromount-G, Invitrogen). Slides were allowed to dry for 24 h in the dark. Fluorescence images were acquired using a Leica DM6000 epifluorescence microscope and hemorrhagic core and perihematomal regions were analyzed using ImageJ v1.53 (NIH).

**Assessment of Neuronal Degeneration**

Fluoro-Jade C (FJC) staining was quantified in one 10-µm section at each of three predefined stereotaxic levels per animal (three sections per animal in total): the collagenase injection level (+0.5 mm from Bregma), 600 µm rostral to the injection site (+1.1 mm from Bregma), and 600 µm caudal to the injection site (−0.1 mm from Bregma). These predefined levels were used to provide standardized regional sampling across animals. FJC-positive cellular profiles were quantified using a DAPI-anchored analysis in ImageJ v1.53 (NIH). DAPI-positive nuclei were first automatically segmented using the Analyze Particles function with a minimum particle area of 10 µm² and a circularity range of 0–1, with no additional circularity-based exclusion criterion. The resulting DAPI-defined regions of interest (ROIs) were added to the ROI Manager. For each image, background FJC fluorescence was estimated from the area outside the DAPI-positive ROIs using the median fluorescence intensity. For each DAPI-defined ROI, the median FJC fluorescence intensity was measured, and a background-corrected FJC signal was calculated as the median FJC intensity within the ROI minus the median background intensity. A DAPI-positive profile was classified as FJC-positive when its background-corrected FJC signal exceeded an image-specific positivity threshold defined as 1.645 × the standard deviation of the background fluorescence. Results were expressed as the density of DAPI+/FJC+ profiles per mm² and as the percentage of DAPI-positive profiles classified as FJC-positive. DAPI-positive profile density was also quantified independently.

**Intravital microscopy**

Intravital fluorescence microscopy was performed using the cremaster muscle microcirculation in anesthetized mice. Body temperature was maintained at 37 °C, and the cremaster muscle was exteriorized and continuously superfused with a temperature-controlled buffered saline solution. Microvessels were imaged using an Olympus AX61WI microscope equipped with a 60×/0.9 NA water-immersion objective and a Cooke Sensicam CCD camera. Fluorescently labeled Ir-CPI (Ir-CPI-A488) was visualized together with Ly6G⁺ neutrophils labeled with PE-conjugated anti-Ly6G antibody.

**Randomization, Blinding, Exclusions and Sample Size**

In Cohort A, animals were allocated to experimental groups before experimentation based on body weight. Treatment groups were coded, and image analyses were performed by investigators blinded to group allocation. In Cohort B, animals were allocated to experimental groups based on body weight to ensure balanced weight distribution. Allocation was performed the day before surgery, and block randomization was used to determine the order of stroke induction. To minimize operator-related bias, half of the animals in each group underwent ICH induction by each of the two surgeons involved in the study. Surgeons and investigators responsible for image and data analyses and behavioral assessments were blinded to group allocation.

Predefined exclusion criteria included technical complications, unsuccessful ICH induction and mortality. In Cohort B, animals with a baseline Bederson score > 0 were excluded from neurological outcome analyses. In Cohort A, 34 animals were eligible; 15 were excluded because of technical issues (n = 7) or death (n = 8), resulting in final group sizes of PBS (n = 7), Ir-CPI (n = 6) and enoxaparin (n = 6). For the three groups retained in the present Cohort B analyses, 69 animals were eligible; 10 were excluded because of technical issues (n = 6) or death (n = 4), resulting in 59 animals: sham (n = 19), PBS (n = 20) and Ir-CPI (n = 20). Fisher’s exact test detected no significant difference in exclusion rates between these groups. The enoxaparin group from the underlying Cohort B study was not included in the present longitudinal behavioral analyses because of the early hemorrhagic signal observed in Cohort A. Sample size justification was reported in accordance with the ARRIVE 2.0 guidelines [3]. Group sizes were based on historical data demonstrating statistically detectable differences in neurobehavioral outcomes using similar sample sizes, while accounting for predefined exclusion criteria and anticipated attrition.

**References**

1.	Depreux, F., D. Whitlon, and C.P. Richter, ALZET pump implantation in mice for chronic drug delivery to the cochlea. Sci Rep, 2025. 15(1): p. 36379.

2.	McAteer, M.A., et al., In vivo magnetic resonance imaging of acute brain inflammation using microparticles of iron oxide. Nat Med, 2007. 13(10): p. 1253-8.

3.	Percie du Sert, N., et al., The ARRIVE guidelines 2.0: updated guidelines for reporting animal research. BMJ Open Sci, 2020. 4(1): p. e100115.
