# Supplementary material for: Ixodes ricinus‐Contact Phase Inhibitor Promotes Functional Recovery and Attenuates Neutrophil Associated Neuroinflammation After Experimental Intracerebral Hemorrhage
Source: CNS Neurosci Ther. 2026 Oct 30;32(11):e71227. doi: 10.1002/cns.71227 (PMC13722891; doi:10.1002/cns.71227)
Supplement: Supplementary file 2 [file CNS-32-e71227-s003.docx]

| **Antibody** | **Fluorophore** | **Supplier** | **Catalogue/reference** | **Clone** |
| --- | --- | --- | --- | --- |
| CD45 | BUV395 | BD Biosciences | 563791 | HI30 |
| CD20 | BUV737 | BD Biosciences | 612849 | 2H7 |
| CD123 | BV421 | BD Biosciences | 567279 | 6H6 |
| CD16 | BV605 | BD Biosciences | 563173 | 3G8 |
| CD14 | BV785 | BD Biosciences | 563699 | M5E2 |
| CD3 | FITC | BD Biosciences | 564561 | SK7 |
| Siglec-8 | PE | BioLegend | 347103 | 7C9 |
| HLA-DR | PE-CF594 | BD Biosciences | 562331 | G46-6 |
| CD56 | PE-Vio700 | Miltenyi Biotec | 130-113-875 | REA196 |
| Anti-Ir-CPI | AF647 | Bioxodes, custom reagent | N/A | N/A |

**Table S1. Antibodies used in flow cytometry to assess the association of Ir-CPI with human neutrophils.**

| **Group** | **Inflammatory marker** | **Spearman r (ρ)** | **P value (two‑tailed)** | **n** |
| --- | --- | --- | --- | --- |
| **PBS** | Mean MPO⁺ cells/mm² | 0.29 | 0.56 | 7 |
|  | Mean Ly6G⁺ cells/mm² | 0.18 | 0.71 | 7 |
|  | Mean Ly6G⁺H3Cit⁺MPO⁺ cells/mm² | 0.68 | 0.11 | 7 |
| **Ir‑CPI** | Mean MPO⁺ cells/mm² | -0.43 | 0.42 | 6 |
|  | Mean Ly6G⁺ cells/mm² | -0.49 | 0.36 | 6 |
|  | Mean Ly6G⁺H3Cit⁺MPO⁺ cells/mm² | -0.03 | >0.99 | 6 |
| **Enoxaparin** | Mean MPO⁺ cells/mm² | -0.54 | 0.30 | 6 |
|  | Mean Ly6G⁺ cells/mm² | 0.20 | 0.71 | 6 |
|  | Mean Ly6G⁺H3Cit⁺MPO⁺ cells/mm² | 0.67 | 0.16 | 6 |

**Table S2. Exploratory correlation analyses between lesion volume and inflammatory markers on Day 3 following collagenase‑induced intracerebral hemorrhage in PBS‑, Ir‑CPI‑, and enoxaparin‑treated mice.**

Correlations were assessed at the animal level using Spearman’s rank correlation due to limited sample sizes and the non‑Gaussian distribution of inflammatory markers. The inflammatory data represent the mean of values collected at the injection site and at the rostral and caudal levels.

| **Group** | **Markers** | **Spearman r (ρ)** | **P value (two‑tailed)** | **n** |
| --- | --- | --- | --- | --- |
| **PBS** | Mean Ly6G⁺ cells/mm² vs mean DAPI⁺/FJC⁺ profiles (%) | 0.29 | 0.56 | 7 |
|  | Mean Ly6G⁺H3Cit⁺MPO⁺ cells/mm² vs mean DAPI⁺/FJC⁺ profiles (%) | 0.46 | 0.30 | 7 |
|  | Mean MPO⁺ cells/mm² vs mean DAPI⁺/FJC⁺ profiles (%) | −0.04 | 0.96 | 7 |
| **Ir-CPI** | Mean Ly6G⁺ cells/mm² vs mean DAPI⁺/FJC⁺ profiles (%) | 0.14 | 0.80 | 6 |
|  | Mean Ly6G⁺H3Cit⁺MPO⁺ cells/mm² vs mean DAPI⁺/FJC⁺ profiles (%) | 0.21 | 0.73 | 6 |
|  | Mean MPO⁺ cells/mm² vs mean DAPI⁺/FJC⁺ profiles (%) | −0.43 | 0.42 | 6 |
| **Enoxaparin** | Mean Ly6G⁺ cells/mm² vs mean DAPI⁺/FJC⁺ profiles (%) | 0.31 | 0.56 | 6 |
|  | Mean Ly6G⁺H3Cit⁺MPO⁺ cells/mm² vs mean DAPI⁺/FJC⁺ profiles (%) | 0.32 | 0.54 | 6 |
|  | Mean MPO⁺ cells/mm² vs mean DAPI⁺/FJC⁺ profiles (%) | 0.14 | 0.80 | 6 |

**Table S3. Exploratory correlations between inflammatory markers and neuronal degeneration on Day 3 following collagenase-induced intracerebral hemorrhage in PBS-, Ir-CPI-, and enoxaparin-treated mice.**

Exploratory Spearman rank correlation analyses were performed at the animal level to assess the relationship between inflammatory cell burden and neuronal degeneration on Day 3 post-ICH. Inflammatory marker values represent the mean of measurements obtained at the injection site and at the predefined rostral and caudal levels. Neuronal degeneration was represented by the mean percentage of DAPI-positive profiles classified as FJC-positive across the same three levels using the revised DAPI-anchored quantification method. Analyses were conducted separately for each treatment group. Two-tailed exact P values are reported. None of the correlations reached statistical significance.
