# Supplementary material for: Ixodes ricinus‐Contact Phase Inhibitor Promotes Functional Recovery and Attenuates Neutrophil Associated Neuroinflammation After Experimental Intracerebral Hemorrhage
Source: CNS Neurosci Ther. 2026 Oct 30;32(11):e71227. doi: 10.1002/cns.71227 (PMC13722891; doi:10.1002/cns.71227)
Supplement: Supplementary file 3 [file CNS-32-e71227-s004.docx]

**Figure S1. VCAM‑1-associated endothelial activation in the brain on Day 3 following collagenase‑induced intracerebral hemorrhage in PBS‑, Ir‑CPI‑ and enoxaparin‑treated mice.**

(a) Quantification of VCAM-1-associated MPIO signal volume. Data are presented as box-and-whisker plots showing the median (line), interquartile range (box) and minimum and maximum values (whiskers), and individual values. (b) Representative T2*-weighted MR images acquired before (Pre-MPIO) and after (Post-MPIO) intravenous administration of anti-VCAM-1-conjugated MPIOs in PBS, Ir-CPI-, and enoxaparin-treated mice. Binding of VCAM-1-targeted MPIOs to activated endothelium produces focal hypointense signal voids on T2*-weighted images.

**Figure S2. Rostrocaudal distribution of the hemorrhage on Day 3 after ICH.**

(a) Hemorrhagic area measured across the 20 serial coronal T2*-weighted MRI slices acquired on Day 3. Lines represent the median hemorrhagic area at each slice for PBS-, Ir-CPI-, and enoxaparin-treated mice. (b) Rostrocaudal hemorrhage extent, calculated from the first to the last MRI slice containing detectable hemorrhagic signal and converted to millimeters based on the 0.5-mm slice thickness. No significant difference was observed among groups. (c) Area-weighted centroid of the hemorrhage along the serial MRI stack. No significant difference was observed among groups. Individual animals are shown together with median and interquartile ranges. PBS, n = 7; Ir-CPI, n = 6; enoxaparin, n = 6.
